# Is there a danger of “biocreep” with non-inferiority trials?

**DOI:** 10.1186/1745-6215-12-S1-A29

**Published:** 2011-12-13

**Authors:** Primrose Beryl, Werner Vach

**Affiliations:** 1 Clinical Epidemiology Group, Department of Medical Biometry and Statistics, University Medical Center Freiburg, Germany

## Background

Non-inferiority (NI) trials test a hypothesis that a new treatment is inferior to standard treatment only to a negligible degree. Bio-creep basically refers to the cyclical phenomenon where a slightly inferior treatment becomes the active control for the next generation of NI trials which over time leads to degradation of the efficacy of the investigational treatment [1,2]. We studied the effect estimates from an unselected set of all the registered non-inferiority trials conducted within a seven-year period. The aim was to determine the pre and post trial distribution of the true effect in NI trials from this data using meta-analytic methods.

## Methods

We did a search for all NI trials registered in the National Library of Medicine (NLM)’s Clinical trials register [http://www.ClinicalTrials.gov] [3] which was carried out between January 2000 and December 2007. Trials studying non-inferiority of efficacy as the primary objective were only included. We did a search for information regarding the primary results from these trials in the following steps: the NLM website [3], The Pharmaceutical Research and Manufacturers of America (PhRMA) [4], the International Federation of Pharmaceutical Manufacturers and Associations (IFPMA) website [5] and Pubmed. Web-based search engines and personal communication were also used. Using the retrieved study results, a descriptive and exploratory analysis of the study characteristics and a meta-analysis of the effect estimates were performed using STATA 11 [6].

## Results

Of the 113 registered NI trials, 83 met the inclusion and exclusion criteria. The final results were available for 63 of the 74 completed studies with result estimates with the help of NLM website-44, PhRMA-2, Pharmaceutical websites-7, Pubmed-18 and others-3. The final results were available for 63 of the 74 completed studies. The source of the study results and effect estimates were 53 scientific journal articles, 13 clinical study reports, 4 press releases and 4 reported on the register records. We intend to present the distribution of true effect of NI trials derived based on the above estimates.

## Conclusion

We found a very high likelihood of retrieving results from registered clinical trials making it possible to calculate the pre-study distribution of the true effect in non-inferiority trials. The unanticipated finding of a positive average effect estimate suggests that a decline in standard treatment effect (biocreep) is not imminent, at least on average. However, the intimidating risk of approval of treatments with true negative effects reiterates the need for a careful choice of the margin in NI trials.
